# Rumen methanogen and protozoal communities of Tibetan sheep and Gansu Alpine Finewool sheep grazing on the Qinghai–Tibetan Plateau, China

**DOI:** 10.1186/s12866-018-1351-0

**Published:** 2018-12-13

**Authors:** Jinqiang Huang, Yongjuan Li

**Affiliations:** 1 College of Animal Science and Technology, Gansu Agricultural University, Lanzhou, 730070 China; 2 College of Science, Gansu Agricultural University, Lanzhou, 730070 China

**Keywords:** Rumen protozoa, Methanogen, Community structure, Plateau sheep

## Abstract

**Background:**

Tibetan sheep (TS) and Gansu Alpine Finewool sheep (GS) are both important plateau sheep raised and fed on the harsh Qinghai–Tibetan Plateau, China. Rumen methanogen and protozoal communities of plateau sheep are affected by their hosts and living environments, and play important roles in ruminant nutrition and greenhouse gas production. However, the characteristics, differences, and associations of these communities remain largely uncharacterized.

**Results:**

The rumen methanogen and protozoal communities of plateau sheep were investigated by 16S/18S rRNA gene clone libraries. The predominant methanogen order in both sheep species was Methanobacteriales followed by Methanomassiliicoccales, which is consistent with those seen in global ruminants. However, the most dominant species was *Methanobrevibacter millerae* rather than *Methanobrevibacter gottschalkii* seen in most ruminants. Compared with GS and other ruminants, TS have more exclusive operational taxonomic units and a lower proportion (64.5%) of *Methanobrevibacter*. The protozoa were divided into Entodiniomorphida and Vestibuliferida, including nine genera and 15 species. The proportion of holotrich protozoa was much lower (1.1%) in TS than ordinary sheep. The most predominant genus was *Entodinium* (70.0%) in TS and *Enoploplastron* (48.8%) in GS, while the most common species was *Entodinium furca monolobum* (43.9%) and *Enoploplastron triloricatum* (45.0%) in TS and GS, respectively; *Entodinium longinucleatum* (22.8%) was only observed in TS. LIBSHUFF analysis indicated that the methanogen communities of TS were significantly different from those of GS, but no significant differences were found in protozoal communities.

**Conclusion:**

Plateau sheep have coevolved with unique rumen methanogen and protozoal communities to adapt to harsh plateau environments. Moreover, the host appears to have a greater influence on rumen methanogen communities than on rumen protozoal communities. The observed associations of methanogens and protozoa, together with the findings of previous studies on methane emissions from ruminant livestock, revealed that the lower proportion of *Methanobrevibacter* and holotrich protozoa may be responsible for the lower methane emission of TS. These findings facilitate our understanding of the rumen microbial ecosystem in plateau sheep, and could help the development of new strategies to manipulate rumen microbes to improve productivity and reduce the emission of greenhouse gases.

**Electronic supplementary material:**

The online version of this article (10.1186/s12866-018-1351-0) contains supplementary material, which is available to authorized users.

## Background

The rumen is crucial for feed digestion and energy supply in ruminants. It contains a complex microbial ecosystem including bacteria, methanogens, protozoa, fungi, and bacteriophages. These different types of symbiotic microorganisms interact with one another and affect the host’s digestion and performance. During microbial fermentation and feed energy utilization, methane (CH_4_) is produced as a by-product of methanogenesis by methanogens belonging to the archaeal phylum *Euryarchaeota*. Methane is a notorious greenhouse gas (GHG) with a global warming potential 28-fold higher than CO_2_; it also represents a loss of gross energy intake from 8 to 13% [1, 2]. As an important GHG, CH_4_ from ruminants accounts for 25–40% of the anthropogenic release of CH_4_, of which 90% is derived from rumen microbial methanogenesis [3, 4]. Mitigating CH_4_ released by ruminants would therefore benefit the environment and may increase the efficiency of livestock production.

Methanogenic archaea produce methane mainly by converting the H_2_ and CO_2_ that arise from bacterial fermentation, ciliate protozoa, and aerobic fungi in the rumen [5, 6]. Many factors affect CH_4_ production, including the intake, type and quality of feed, environmental stresses, pH, volatile fatty acids, and the animal species [7, 8]. Measures of CH_4_ mitigation emissions from livestock have been reviewed by Kumar, and two main areas of intervention in the form of dietary and microbial changes are discussed comprehensively [9]. Although these strategies can reduce GHG emissions to a certain extent, for example by limiting chemical inhibitor toxicity to ruminants and decreasing CH_4_ production by dietary manipulation, they nevertheless have a number of disadvantages.

Rumen protozoa are present in the rumen of most domesticated ruminants and play key roles in the digestion and fermentation of feed components. H_2_ is an end product of carbohydrate fermentation by protozoa, but it inhibits their metabolism if it is not removed. Methanogens can, however, utilize H_2_ so form symbiotic relationships with protozoa. This relationship can generate up to 37% of the rumen CH_4_ emission [10]. Defaunation (the removal of protozoa from the rumen) has been investigated as an emission reduction measure, but the results were not consistent [6, 11–14]. Moreover, a successful methane mitigation strategy requires a thorough understanding of the rumen microbial ecosystem and their associations.

The Qinghai–Tibetan Plateau (QTP) in China is one of the major drivers of global climatic conditions, and offers the most extreme environments (cold, strong UV radiation, low oxygen, high altitude, and poor forage resources) for the survival of ruminant species [15]. After a long period of evolution, Tibetan sheep (TS; *Ovis aries*) as one of the major indigenous ruminants have adapted to live in the harsh environment of the plateau and provide sustenance and income for Tibetan pastoralists. TS graze on natural pasture with coarse grasses as their only food, and have developed particular physiologies and nutrition mechanisms to survive. The Gansu Alpine Finewool sheep (GS) is an introduced domestic ruminant species (a crossbred sheep from the TS and Xinjiang Finewool sheep) that was bred 30 years ago, and graze under similar extreme conditions with TS on the QTP.

High-altitude ruminants were shown to have rumen microbial ecosystems that differ significantly from their low-altitude relatives and which yield significantly lower levels of methane [16–19]. We previously showed that the rumen bacterial community of TS was distinctly different from that of GS [20]. We hypothesized that indigenous (TS) and introduced ruminants (GS) have coevolved unique rumen methanogen and protozoal communities to adapt to the harsh QTP environments and host differences. Therefore, in the present study, we performed a community analysis of methanogens and protozoa of TS and GS using 16S/18S rRNA gene libraries, and also analyzed the differences and associations of methanogens and protozoa between indigenous and introduced ruminants under the same altitude environment conditions. To our knowledge, the characteristics, differences, and associations in communities of methanogens and protozoa have not been systematically investigated in TS and GS under QTP grazing conditions. Our findings will contribute to an understanding of the rumen microbial ecosystem of plateau sheep, and help manipulate rumen microbes to improve productivity and reduce the emission of greenhouse gases.

## Methods

### Animals and sample collection

All the experimental procedures were approved by the Gansu Agricultural University Animal Welfare and Ethical Committee and were performed in accordance with the Regulations for the Administration of Affairs Concerning Experimental Animals (The State Science and Technology Commission of P. R. China, 1988). This study had no lasting harmful effect on the health of the animals.

Six male TS (aged 2 ± 0.1 years, 42 ± 2 kg) and six male GS (aged 2 ± 0.1 years, 40 ± 2 kg) from a sheep farm on the QTP in China were randomly selected for sampling. Written informed consent was obtained from the owner for the involvement of their sheep in our study. The sheep grazed on natural alpine meadow grasslands (at an altitude of 3000–3300 m above sea level) comprising grasses and sedges. The main grass species were *Roegnevia kamoji*, *Koeleria litwinowi*, and *Stipa aliene*, the cellulose of them ranging 30.34–42.06% and crude protein ranging 8.56–10.39% of dry matter. The dominant sedge species were *Kobresia capillifolia*, *Carex atrofusca*, and *K. pygmaea*, with cellulose and crude protein ranged 22.58–33.25% and 10.45–13.46% of dry matter, respectively. The sheep herds were maintained outdoors and have the herbage of above grass and sedge species as the exclusive feed until sampling in autumn. Approximately 30 ml rumen digesta was extracted from each sheep using esophageal tubing attached to an electric pump and squeezed through four layers of sterilized cheesecloth. The filter fluid fraction was transferred immediately into three 2-mL sterile tubes and stored at − 80 °C until DNA extraction.

### DNA extraction and PCR amplification

Total genomic DNA was extracted as previously described with minor modifications [20]. In brief, the rumen fluid was centrifuged in 1.5-ml microcentrifuge tube at 6,000 g for 2 min and resuspended in 500 μl 1 × TE buffer and 30 μl of 20 mg/ml proteinase k, followed by incubation at 37 °C for 1 h. Following incubation, 5 M NaCl were added and mixed before the addition of CTAB/NaCl. The lysate was completely mixed and incubated at 65 °C for 10 min. The cell lysates were extracted with chloroform-isoamyl alcohol (24:1) and phenol-chloroform-isoamyl alcohol (25:24:1) before nucleic acids were precipitated with 0.6 volume isopropanol. DNA was collected by centrifugation, washed with 70% ethanol, resuspended in TE buffer. DNA was stored at − 80 °C prior to the amplification of 16S/18S rRNA. 16S rRNA genes were amplified using the methanogen-specific primers Met86F (5′-GCTCAGTAACACGTGG-3′) and Met1340R (5′-CGGTGTGTGCAAGGAG-3′) [21]. The 18S rRNA gene was amplified using the protozoa-specific primer P-SSU-342f (5′-CTTTCGATGGTAGTGTATTGGACTAC-3′) and reverse primer Medlin B (5′-TGATCCTTCTGCAGGTTCACCTAC-3′) [22, 23]. PCR amplification was performed according to the following program: denaturation at 94 °C for 5 min; followed by 25 cycles of denaturation at 94 °C for 45 s, annealing at 55 °C for 45 s, and elongation at 72 °C for 2 min, then a final extension at 72 °C for 10 min. PCR products were purified using a Gel Extraction Kit and ligated into pMD18-T simple vectors (Takara Bio, Dalian, China). Hybrid vectors were transformed into *Escherichia coli* DH5α. Approximately 200 white colonies from a single Luria–Bertani plate were selected for restriction fragment length polymorphism analysis. Cloned 16S/18S rRNA genes were reamplified by PCR using the plasmid primers RV-M and M13–47, which bind to sites next to the 16S/18S rRNA gene. The PCR products were respectively digested with *Hae* III, *Alu* I, and *Hpa* II. Digested fragments were separated by electrophoresis on 4.0% agarose gels and compared to identify redundant clones.

### Sequence and phylogenetic analysis

The represented distinctive clones were selected and sequenced in both directions with an ABI 3730 DNA automatic sequencer (Applied Biosystems, Foster City, CA, USA). The sequences were analyzed using the Mallard program to identify and exclude chimeric sequences [24]. Similarities of nonchimera sequences were searched in the GenBank database using the BLAST program. Valid sequences were grouped into operational taxonomic units (OTUs) at a 0.98 similarity threshold using the MOTHUR program [25, 26]. Sequences were aligned using ClustalX (version 2.0), and phylogenetic trees were constructed by PHYLIP (version 3.69) [27, 28].

### Statistical analysis

Coverage (*C*) of each library was calculated according to the equation: *C* = [1 − (*n* / *N*)] × 100, where *N* is the total number of clones in the library and *n* is the number of unique OTUs that occurred only once in the clone library [20]. The frequency (*F*) was calculated as follows: *F* = (*m* / *N*) × 100, where *m* is the number of clones of an OTU in a library and *N* is the total number of clones in the same library. Rarefaction analysis of library structure was conducted using Analytic Rarefaction [20]. Diversity indices, such as Shannon–Weiner *H*, abundance-based coverage estimator (*S*_ACE_), and bias-corrected Chao1 (*S*_Chao1_) were calculated and used to measure the diversity for each library using MOTHUR [25]. A DNA distance matrix (dnadist) with Jukes-Cantor option was calculated by using the DNADIST program within the PHYLIP software package. The dnadist matrix was used for LIBSHUFF gene library comparison. Differences were considered significantly different when *P* < 0.025 with LIBSHUFF analysis.

### Nucleotide sequence accession numbers

Nucleotide sequences were designed with the prefix TM and GM to represent 16S rRNA gene sequences from TS and GS clone libraries, respectively, and TP and GP to represent 18S rRNA gene sequences from TS and GS clone libraries, respectively. All 16S rRNA nucleotide sequences generated from this study have been deposited in GenBank under accession numbers MF787942 to MF788003 and MF788004 to MF788068 for clones obtained from TS and GS, respectively. The 18S rRNA gene partial sequences have been deposited under accession numbers MF995562 to MF995592 and MF995593 to MF995629 for clones obtained from TS and GS, respectively.

## Results

### Methanogen 16S rRNA and protozoal 18S rRNA gene libraries from TS and GS

A total of 138 clones were obtained from the TS methanogen 16S rRNA gene (TM) library, revealing 62 unique sequences that were assigned to 28 OTUs based on the 98% identity criterion (Table 1). OTU1 and OTU2 represented most of the clones at a frequency of 25.4 and 15.9%, respectively (Additional file 1: Table S1). The GS methanogen 16S rRNA gene (GM) library had 155 clones with 65 unique sequences assigned to 18 OTUs (Table 1). OTU1 and OTU2 were the most highly represented clones at 36.8 and 33.5%, respectively. Using a 98% similarity cutoff value, coverage of the clone library was estimated at 92.1% for the TM library and 95.5% for the GM library. Rarefaction curves in the two libraries showed a clear trend toward reaching a plateau (Additional file 2: Figure S1). The results of coverage and the rarefaction curve indicated that both libraries were well sampled for diversity analysis.

**Table 1:** Biodiversity and predicted richness from the rumen content of Tibetan sheep and Gansu Alpine Finewool sheep

Sample	No. of clones	No.of unique sequences^a^	No. of OTUs^b^	*C* (%)	*H* (95%CIs)^c^	*S*_ACE_ (95%CIs)^c^	*S*_Chao1_ (95%CIs) ^c^
TM	138	62	28	92.1	2.8 (2.5, 3.1)	44.1 (39.1, 51.1)	38.7 (30.5, 66.4)
GM	155	65	18	95.5	2.1 (1.8, 2.4)	24.5 (21.7, 28.9)	21.7 (18.0, 39.8)
TP	180	31	10	98.3	2.1 (1.9, 2.4)	10.6 (10.1, 16.1)	10.0 (9.5, 16.4)
GP	169	37	9	98.8	1.5 (1.2, 1.9)	8.9 (8.2, 12.4)	8.5 (8.0, 16.3)

^a^Unique 16S rRNA gene sequences were determined via RFLP analysis

^b^OTUs of the 16S rRNA gene sequences were determined as described in the text. The coverage (*C*), Shannon-Weiner (*H*) indices, and *S*_ACE_ and *S*_Chao1_ richness estimators were calculated with the OTU data

^c^The 95% confidential intervals (95%CIs) were provided when calculating richness estimators

A total of 180 clones were subjected to similarity analysis in GenBank by BLAST analysis in the TS protozoal 18S rRNA gene (TP) library (Table 1). All sequences showed > 97% sequence similarities with those of protozoa available in GenBank (Additional file 3: Table S2). Based on < 98% sequence similarity, the clones were grouped into 10 OTUs. OTU8, OTU1, and OTU2 represented most of the clones at a frequency of 25.6, 20.0, and 20.0%, respectively. In the GS protozoal 18S rRNA gene (GP) library, a total of 169 clones were grouped into 9 OTUs, and OTU1 represented nearly half (48.5%) of the total clones (Table 1; Additional file 3: Table S2). The coverage of the clone library was 98.3% for the TP library and 98.8 for the GP library (Table 1). Clear plateaus were observed in the rarefaction curves from both TP and GP libraries (Additional file 2: Figure S1).

### Sequence and phylogenetic placement analysis

Within the TM library, 67 of 138 clones had ≥98% identity to known species of rumen methanogens, and 43 clones shared 95–98% identity (Additional file 1: Table S1). The most dominant genus (sequence identity ≥95%) in the TM library was *Methanobrevibacter* at a frequency of 64.5%. The most dominant species (sequence identity ≥98%) was *Methanobrevibacter millerae*, accounting for 41.3% (57 clones) of the total clones. A total of 28 clones displayed only 93.5–94.6% sequence identities to *Methanobrevibacter*, *Methanosphaera*, *Candidatus Methanoplasma*, and *Methanosphaera stadtmanae*, so likely represented unknown families of methanogens. In the GM library, 111 of 155 clones shared ≥98% similarity to known sequences of rumen methanogens, and 26 clones shared 95–98% identity (Additional file 1: Table S1). The most dominant genus and species were also *Metharrobrevibacter* and *Methanobrevibacter millerae*, accounting for 85.2% (132 clones) and 71.6% (111 clones) of the total clones, respectively. Only 10 clones had sequence identities < 95%.

In the TP library, 133 of 180 clones shared ≥98% similarity to known sequences of rumen protozoa and the remaining clones shared 97–98% identity (Additional file 3: Table S2). In the GP library, all clones shared ≥98% identity (Additional file 3: Table S2). In the TP library, 178 of 180 clones were identified as belonging to Entodiniomorphida, compared with 164 of 169 clones in the GP library; only two (TP) and five clones (GP) were related to *Dasytricha ruminantium* of the order Vestibuliferida in the two libraries.

**Fig. 1:**
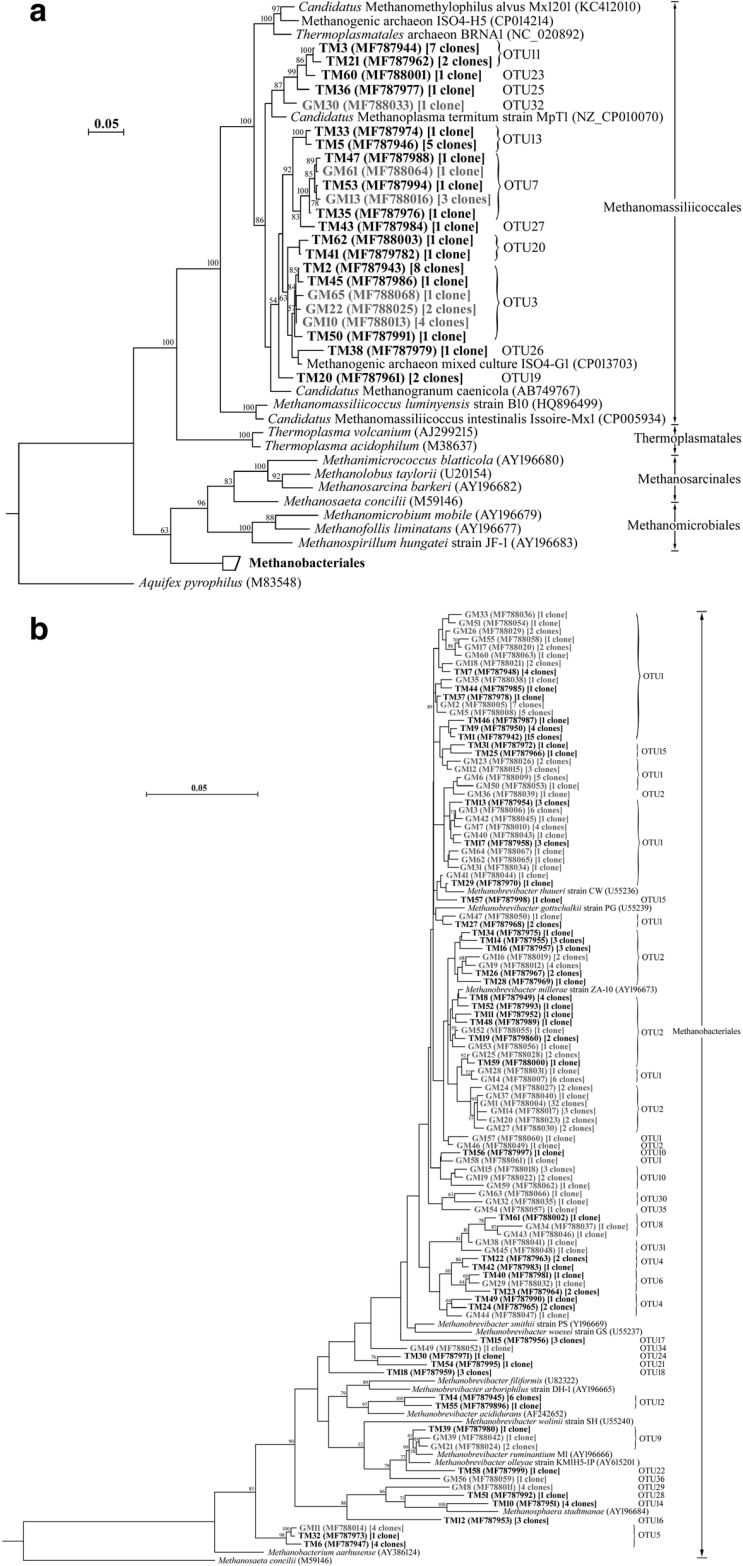
Phylogenetic analysis of methanogen partial 16S rRNA sequences from clone libraries. The phylogenetic tree was constructed using the diatance and neighbor-joining method with the Kimura two-parameter model for nucleotide change. In which, sequences from Methanomassiliicoccales are presented in (**a**), and sequences from Methanobacteriales are presented in (**b**). The scale bar represents 5% estimated sequence divergence. Bootstrap values above 50 (based on 1000 bootstrap resamplings) were indicated. The 16S rRNA gene sequences obtained in this study are shown in bold, and numbers of clones in each OTU are labeled in the brackets. The prefix TM and GM represent methanogen 16S rRNA gene sequences from Tibetan sheep and Gansu Alpine Finewool sheep rumen clone libraries, respectively

**Fig. 2:**
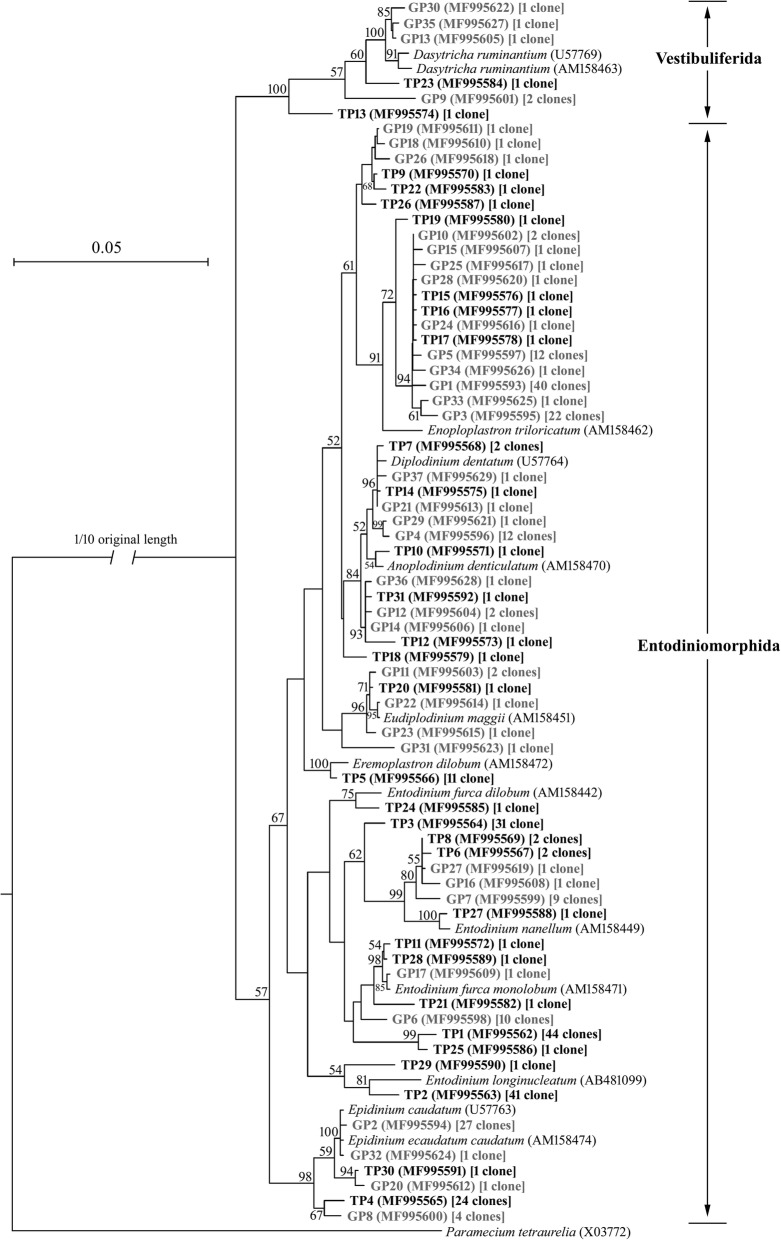
Phylogenetic analysis of protozoal 18S rRNA sequences from Tibetan sheep and Gansu Alpine Finewool sheep clone libraries. The phylogenetic tree was constructed using the diatance and neighbor-joining method with the Kimura two-parameter model for nucleotide change. The scale bar represents 5% estimated sequence divergence. Bootstrap values above 50 (based on 1000 bootstrap resamplings) were indicated. The 18S rRNA gene sequences obtained in this study are shown in bold, and numbers of clones in each OTU are labeled in the brackets. The prefix TP and GP represent protozoal 18S rRNA gene sequences from Tibetan sheep and Gansu Alpine Finewool sheep rumen clone libraries, respectively

Phylogenetic trees were constructed to show the phylogenetic placement and taxonomic relationships of the methanogen sequences from the TM and GM (Fig. 1). Methanogen sequences were grouped with two clades, the Methanomassiliicoccales and Methanobacteriales. OTU1–2, 4–6, 8–10, 12, 14–18, 21–22, 24, 28–31, and 34–36 grouped within the Methanobacteriales and the remaining 11 OTUs represented 48 clones within the Methanomassiliicoccales. Figure 2 shows the results of phylogenetic analysis of 18S rRNA sequences from TP and GP. Rumen protozoa mainly divided into two groups. The first group of seven clones was phylogenetically placed within the order Vestibuliferida, and the other clones were placed within the Entodiniomorphida.

### Comparative analysis between TS and GS

In 16S rRNA libraries, a total of 293 clones were assigned to 36 OTUs, of which 18 OTUs were unique to the TM library and eight OTUs to the GM library. Ten OTUs were common to both libraries. The Shannon–Wiener index analysis indicated a higher diversity among TM (2.8 ± 0.3) compared with GM (2.1 ± 0.3) (Table 1). Richness estimators of *S*_ACE_ and *S*_Chao1_ were also higher among TM (44.1 and 38.7) than GM (24.5 and 21.7) (Table 1), and LIBSHUFF analysis showed that the methanogen community structure of the TS was significantly different from those of the GS.

**Fig. 3:**
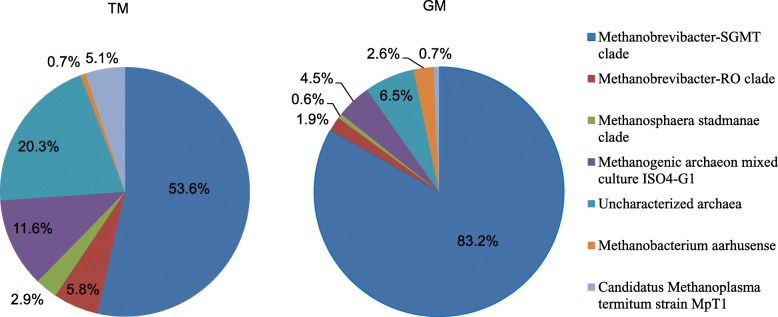
Pie chart representation of methanogen 16S rRNA gene clone distribution. The *smithii*-*gottschalkii*-*milleraethaurei*-*thaurei* (SGMT) clade consists of sequences that phylogenetically group within the major clade consisting of *M. smithii*, *M. gottschalkii*, *M. millerae*, and *M. thaueri*. Similarly, the phylogenetic group consists of *M. ruminantium* and *M. olleyae* sequences are represented in the *ruminantium*-*olleyae* (RO) clade. The TM and GM stand for Tibetan sheep and Gansu Alpine Finewool sheep rumen methanogen 16S rRNA gene libraries, respectively

In recent years, *Methanobrevibacter*-related sequences have generally been divided into two categories [29–31]. The SGMT clade consists of *Methanobrevibacter smithii* (S), *Methanobrevibacter gottschalkii* (G), *Methanobrevibacter millerae* (M), and *Methanobrevibacter thaueri* (T) sequences, while RO includes *Methanobrevibacter ruminantium* (R) and *Methanobrevibacter olleyae* (O) sequences. The distribution of the methanogen 16S rRNA gene clones in the rumen of TS and GS is shown in Fig. 3. The SGMT clade dominated the archaea populations, with GM (83.2%) obviously higher than TM (53.6%). The proportion of sequences belonging to the RO clade was higher in TM (5.8%) than GM (1.9%). Additionally, the relative abundance of the methanogenic archaeon mixed culture ISO4-G1 (Isolation experiment 4-G1) was higher in TM (11.6%) than GM (4.5%).

In 18S rRNA libraries, a total of 349 clones were assigned to 12 OTUs, with OTU1–7 common to TP and GP libraries. OTU8–10 and OTU11–12 were unique to TP and GP, respectively. Shannon–Wiener index analysis and richness estimators of *S*_ACE_ and *S*_Chao1_ were both higher in the TP library compared with the GP library (Table 1), but LIBSHUFF analysis showed that the protozoal community structure between TS and GS was not significantly different.

The distribution of protozoal 18S rRNA gene clones in the rumen of TS and GS is shown in Additional file 4: Table S3. A total of nine genera were observed including *Entodinium*, *Enoploplastron*, *Epidinium*, *Eremoplastron*, *Anoplodinium*, *Diplodinium*, *Polyplastron*, *Eudiplodinium* and *Dasytricha* (Additional file 4: Table S3). *Entodinium* was the most common genus in the TP library, at a frequency of 70.0%, while *Enoploplastron* was most common in the GP library, at 48.8%. Protozoa from the genus *Eremoplastron* were only found in the TP library, and *Polyplastron* was found only in the GP library (Additional file 4: Table S3). A total of 15 species were observed in both libraries, with *Entodinium furca monolobum* the highest proportion at a frequency of 43.9% in the TP library but only 6.5% in the GP library. *Enoploplastron triloricatum* was the most dominant species at a frequency of 45.0% in the GP library but only 2.2% in the TP library. *Entodinium longinucleatum* was only observed in the TP library, at a proportion of 22.8%.

## Discussion

Tibetan sheep (TS) and Gansu Alpine Finewool sheep (GS) are major ruminants that graze on the QTP, and this study analyzed and compared the characteristics, differences, and associations of their rumen methanogen and protozoal communities by 16S/18S rRNA gene clone libraries. The most dominant species was found to be *Methanobrevibacter millerae*, compared with *Methanobrevibacter gottschalkii* in other ruminants. Compared with GS, TS have more exclusive OTUs and a lower proportion of *Methanobrevibacter*. The protozoa were divided into two clades, Entodiniomorphida and Vestibuliferida, which included nine genera and 15 species. The most predominant genus was *Entodinium* in TS and *Enoploplastron* in GS. Protozoa from the genus *Eremoplastron* were only detected in TS and *Polyplastron* were only found in GS. The most prodominant species were *Entodinium furca monolobum* and *Enoploplastron triloricatum* in TS and GS, respectively. These findings suggest that the plateau sheep have coevolved with unique rumen methanogen and protozoal communities to adapt to harsh plateau environments. Moreover, the host appears to have a greater influence on rumen methanogen commnuities but a weaker impact on rumen protozoal commnuities. The lower proportion of *Methanobrevibacter* and holotrich protozoa (Vestibuliferida) may be responsible for the observed lower methane emission in TS.

We found that the most dominant and largest methanogen group was *Methanobrevibacter millerae* in the two 16S RNA libraries. Henderson et al. previously analyzed the rumen microbial community composition of 742 samples from 32 animal species, and revealed *Methanobrevibacter gottschalkii* and *Methanobrevibacter ruminantimum* to be the two largest groups of rumen archaea [32]. Our results are consistent with those of other ruminants at the genus level, but the dominant species were different. Sequences related to *Methanobrevibacter gottschalkii* and *Methanobrevibacter ruminantimum* clades were also detected in the two 16S rRNA libraries, but at a very low frequency. In the sika deer, the *Methanobrevibacter millerae* clade was the most dominant, and it was also found in horses, and Hanwoo cattle [31, 33, 34]. *Methanobrevibacter* was also the predominant rumen methanogen in yaks grazing natural pastures [35]. The methanogenic archaea of yaks grazing on the QTP were dominated by Methanobacteriaceae, followed by Methanomassiliicoccaceae [36]. However, Methanomassiliicoccaceae was the predominant group in TS and crossbred sheep fed ad libitum a diet of oaten hay:barley (70:30) [16]. The reasons for these differences could be the different primers and sequencing methods used, variations in dietary factors, and different hosts [37]. Another dominant rumen archaea group was methanogenic archaeon mixed culture ISO4-G1 which belongs to the order Methanomassiliicoccales [38]. This is a group of relatively poorly-characterized methanogens which could include some as yet unnamed species and genera [39]. In the present study, 11 OTUs representing 48 clones were grouped in the Methanomassiliicoccales, suggesting that a considerable portion of the methanogens remain to be functionally characterized.

The community structures of methanogens in the rumen of TS and GS were clearly different, even though the two species experience the same environmental conditions and diet on the QTP. Higher biodiversity and richness were evident in the TS rumen than that of the GS, with 18 exclusive OTUs in the TS compared with only 10 in the GS. TS as a indigenous species have lived on the QTP for a long time, and have evolved various strategies to adapt to this harsh plateau environment. They therefore possess unique morphological, physiological, and behavioural characteristics, inlcuding the convergent evolution and adaptation of their rumen microbial community structure [18, 32].

The SGMT and RO clades provided greater insights into the characteristics of the *Methanobrevebacter* community. A considerably different SGMT–RO methanogen distribution was observed between the TS and GS groups, with a lower proportion of SGMT methanogens detected in the TS than in the GS. In cows, a higher proportion of SGMT (consisting of *M. smithii*, *M. gottschalkii*, *M. millerae*, and *M. thaueri*) was previously associated with higher methane emission, while lower methane emission and high volatile fatty acid production was detected in TS compared with other sheep [18, 40]. In New Zealand sheep, a positive correlation between methane yield and the relative abundance of *M. gottschalkii* clade were also found by transcriptome analysis [41]. We speculate that the low proportion of SGMT and highly efficient metabolism of TS may be responsible for the decreased methane emission. However, further studies are needed to confirm this.

ISO4-G1 is a methylotrophic methanogen isolated from a sheep rumen, which is widely distributed in different ruminants. The genome of ISO4-G1 has previously been sequenced, and analysis suggested that it relies on hydrogen-dependent methylotrophic methanogenesis to produce energy, using methanol and methylamines as substrates [38]. In this study, the proportion of ISO4-G1 was higher in TS than in GS. In the Chinese goat, the relative abundance of ISO4-G1 was significantly higher in those fed a hay diet than those receiving a high grain diet [42]. The higher proportion of ISO4-G1 observed in TS in the present study implied that ISO4-G1 may help TS adapt to the coarse grasses of the QTP as an energy source. Another *Candidatus Methanoplasma termitum* strain, MPT1, was detected in both plateau sheep, and a much higher proportion was found in TS than in GS. These results are consistent with previous findings [19].

Despite contributing up to 50% of the bio-mass in the rumen, the role of protozoa remains unclear in rumen microbial ecosystem because it is difficult to maintain rumen protozoa in axenic culture [43]. Some researchers believed that protozoa may be not essential to the animal to survive; eliminating them has been suggested as a means of mitigating methane emissions [11, 44]. The average reduction of methane production in the absence of protozoa was around 12% [6, 11], although several studies reported that defaunation had no effect on methane emissions [12–14]. The reasons for this controversy are unclear, but a greater understanding of protozoal communities should aid the reduction of methane production by manipulating their numbers and structure. Compared with methanogens, little is known about rumen protozoa. In the present study, we estimated that protozoa had a reduced species richness in the rumen compared with bacteria and methanogen in TS and GS, suggesting that the abundance and diversity of eukaryotes were lower than that of prokaryotes [20].

Eight genera of protozoa were previously observed in TS in Nyingchi (China), including *Entodinium*, *Epidinium*, *Diplodinium*, *Polyplastron*, *Eudiplodium*, *Isotricha*, *Ostracodinium*, and *Ophryoscoex*; *Entodinium* was the most abundant [45]. All of these, except *Isotricha*, *Ostracodinium*, and *Ophryoscoex*, were detected in our study, although *Entodinium* dominated in TS and *Enoploplastron* in GS. *Entodinium* was also the most predominant genus in another grazing Mongolian sheep, accounting for 82.6% of all species [46], as well as in a study by Guirong et al. in the rumen of yak in Tibet, Sichuan, and Inner Mongolia (51.9–61.0%) [47]. These results implied that *Entodinium* may be an important protozoan of Mongolian sheep and yak although its function is not clear [43]. *Enoploplastron triloricatum*, seen in GS in our study, produces cellulase and grows well in vitro on dried grass alone, while *Enoploplastron stokyi* is another species of the same genus [48, 49]. The observed differences in the dominant protozoal genera between TS and GS are likely to reflect inter-species differences because both sheep have the same diet.

Guyader et al. [50] reported a significant linear relationship between protozoal concentration and methane emissions. Moreover, a meta-analysis showed that the elimination of ciliate protozoa not only reduced methane production by up to 11% but also increased the microbial protein supply by up to 30% [43]. Tymensen et al. investigated the structures of free-living and protozoa-associated methanogen communities (PAM) in foraged cattle, and found that *Methanobrevibacter* species were more abundant in PAM but *Methanomicrobium* species prodominated in free-living communities [51], while a higher relative abundance of *Methanobrevibacter* was associated with high methane production [41]. Members of the *Methanobrevibacter* (accounting for 74% of all archaea) were found in almost all rumen samples of 32 animal species, and were the largest group [32]. In comparison, the relatively lower proportion of *Methanobrevibacter* (64.5%) in TS of our study may be responsible for their lower methane emission. Furthermore, different rumen protozoa have different impacts on methanogenesis, with holotrich protozoa being more associated with methanogenesis while entodiniomorphids play a key role in the bacterial protein turnover [41]. Holotrich protozoa account for around 4.2% of rumen protozoa in ordinary sheep [52], compared with only 1.1% in TS in the present study, which could also explain the lower methane emission.

## Conclusions

The rumen methanogen and protozoal communities of TS and GS grazing on the QTP were described and compared in the present study, and shown to differ from those of other ruminants. The diversity of methanogen communities differed significantly between TS and GS, indicating that between-species differences affect rumen methanogen community structures. However, protozoal communities were only slightly different from those of other ruminants and not significantly different between TS and GS, demonstrating that they are relatively stable among species and between environments. The associations of methanogens and protozoa revealed that the lower proportion of *Methanobrevibacter* and holotrich protozoa in TS may be responsible for their lower methane emissions. This study facilitates our understanding of the rumen microbial ecosystem in plateau sheep which may help to explain their lower rates of methanogenesis compared with ordinary sheep. However, our study was limited by its lack of methane emission data, so further studies are necessary to measure methane emissions and to explore how rumen methanogens and protozoa can be manipulated to improve productivity and reduce methane production.

## Additional files

Additional file 1: **Table S1.** Similarity values of rumen methanogens from Tibetan sheep and Gansu Alpine Finewool sheep from Qinghai-Tibetan Plateau, China. (PDF 65 kb)

Additional file 2: **Figure S1.** Rarefaction curves generated for OTUs of 16S rRNA and 18S rRNA gene clones. The TM and GM stand for Tibetan sheep and Gansu Alpine Finewool sheep rumen methanogen 16S rRNA gene libraries, respectively. The TP and GP stand for Tibetan sheep and Gansu Alpine Finewool sheep rumen protozoal 18S rRNA gene libraries, respectively. (PDF 21 kb)

Additional file 3: **Table S2.** Similarity values of rumen protozoa from Tibetan sheep and Gansu Alpine Finewool sheep from Qinghai-Tibetan Plateau, China. (PDF 52 kb)

Additional file 4: **Table S3.** Genera of protozoa in the ciliate protozoan population of the Tibetan sheep and Gansu Alpine Finewool sheep rumen. (PDF 16 kb)

## Abbreviations

DNA: Deoxyribonucleic acid
GHG: Greenhouse gas
GM: GS methanogen 16S rRNA gene
GP: GS protozoal 18S rRNA gene
GS: Gansu Alpine Finewool sheep
OTU: Operational taxonomic units
PCR: Polymerase chain reaction
QTP: Qinghai–Tibetan Plateau
TM: TS methanogen 16S rRNA gene
TP: TS protozoal 18S rRNA gene
TS: Tibetan sheep
