# Supplementary material for: Rumen methanogen and protozoal communities of Tibetan sheep and Gansu Alpine Finewool sheep grazing on the Qinghai–Tibetan Plateau, China
Source: BMC Microbiol. 2018 Dec 13;18:212. doi: 10.1186/s12866-018-1351-0 (PMC6293568; doi:10.1186/s12866-018-1351-0)
Supplement: Supplementary file 1 — Table S1. Similarity values of rumen methanogens from Tibetan sheep and Gansu Alpine Finewool sheep from Qinghai-Tibetan Plateau, China. (PDF 65 kb) [file 12866_2018_1351_MOESM1_ESM.pdf]

Table S1 Similarity values of rumen methanogens from Tibetan sheep and Gansu Alpine Finewool sheep from Qinghai-Tibetan Plateau, China

OTU	Phylotype[a] (Clones[b])		Nearest valid taxon	Seq%[c]	
	TM[d]	GM[d]		TM	GM
1	TM1 (15)	GM2 (7)	*Methanobrevibacter millerae* strain ZA-10	99.0	98.7
1	TM7 (4)	GM3 (6)	*Methanobrevibacter millerae* strain ZA-10	98.2	98.7
1	TM9 (4)	GM4 (6)	*Methanobrevibacter millerae* strain ZA-10	98.7	98.7
1	TM13 (3)	GM5 (5)	*Methanobrevibacter millerae* strain ZA-10	98.6	98.4
1	TM17 (3)	GM6 (5)	*Methanobrevibacter millerae* strain ZA-10	98.5	98.7
1	TM27 (2)	GM7 (4)	*Methanobrevibacter millerae* strain ZA-10	98.2	98.6
1	TM29 (1)	–	*Methanobrevibacter thaueri* strain CW	98.6	–
1	TM37 (1)	GM12 (3)	*Methanobrevibacter millerae* strain ZA-10	98.6	98.5
1	TM44 (1)	GM17 (2)	*Methanobrevibacter millerae* strain ZA-10	98.4	98.4
1	–	GM18 (2)	*Methanobrevibacter gottschalkii* strain PG	–	97.9
1	TM46 (1)	GM23 (2)	*Methanobrevibacter millerae* strain ZA-10	98.9	98.1
1	–	GM26 (2)	*Methanobrevibacter millerae* strain ZA-10	–	98.4
1	–	GM28 (1)	*Methanobrevibacter millerae* strain ZA-10	–	98.8
1	–	GM31 (1)	*Methanobrevibacter millerae* strain ZA-10	–	98.8
1	–	GM33 (1)	*Methanobrevibacter millerae* strain ZA-10	–	98.0
1	–	GM35 (1)	*Methanobrevibacter millerae* strain ZA-10	–	98.5
1	–	GM40 (1)	*Methanobrevibacter millerae* strain ZA-10	–	98.6
1	–	GM41 (1)	*Methanobrevibacter millerae* strain ZA-10	–	98.5
1	–	GM42 (1)	*Methanobrevibacter millerae* strain ZA-10	–	98.4
1	–	GM47 (1)	*Methanobrevibacter millerae* strain ZA-10	–	98.5
1	–	GM50 (1)	*Methanobrevibacter millerae* strain ZA-10	–	98.2
1	–	GM51 (1)	*Methanobrevibacter millerae* strain ZA-10	–	98.1
1	–	GM55 (1)	*Methanobrevibacter millerae* strain ZA-10	–	97.8
1	–	GM57 (1)	*Methanobrevibacter millerae* strain ZA-10	–	98.7
1	–	GM58 (1)	*Methanobrevibacter gottschalkii* strain PG	–	98.1
1	–	GM62 (1)	*Methanobrevibacter millerae* strain ZA-10	–	98.9
1	–	GM60 (1)	*Methanobrevibacter millerae* strain ZA-10	–	98.1
1	–	GM64 (1)	*Methanobrevibacter millerae* strain ZA-10	–	98.9
2	TM8 (4)	GM1 (32)	*Methanobrevibacter millerae* strain ZA-10	99.4	98.9
2	TM11 (3)	GM9 (4)	*Methanobrevibacter millerae* strain ZA-10	98.9	98.6
2	TM14 (3)	GM14 (3)	*Methanobrevibacter millerae* strain ZA-10	98.3	98.5
2	TM16 (3)	GM16 (2)	*Methanobrevibacter millerae* strain ZA-10	98.3	98.4
2	–	GM20 (2)	*Methanobrevibacter smithii* strain PS	–	97.2
2	TM19 (2)	GM24 (2)	*Methanobrevibacter millerae* strain ZA-10	99.1	98.7
2	TM26 (2)	GM25 (2)	*Methanobrevibacter millerae* strain ZA-10	99.0	99.1
2	TM28 (1)	GM27 (2)	*Methanobrevibacter millerae* strain ZA-10	98.1	98.8
2	TM34 (1)	GM36 (1)	*Methanobrevibacter millerae* strain ZA-10	98.8	98.7
2	TM48 (1)	GM37 (1)	*Methanobrevibacter millerae* strain ZA-10	99.2	98.7
2	TM52 (1)	GM46 (1)	*Methanobrevibacter millerae* strain ZA-10	99.4	99.4

2	TM59 (1)	GM52 (1)	*Methanobrevibacter millerae* strain ZA-10	99.1	99.2
2	–	GM53 (1)	*Methanobrevibacter millerae* strain ZA-10	–	98.8
3	TM2 (8)	GM10 (4)	Methanogenic archaeon mixed culture ISO4-G1	98.2	98.3
3	TM45 (1)	GM22 (2)	Methanogenic archaeon mixed culture ISO4-G1	97.6	97.8
3	TM50 (1)	GM65 (1)	Methanogenic archaeon mixed culture ISO4-G1	97.9	97.4
4	TM22 (2)	GM44 (1)	*Methanobrevibacter millerae* strain ZA-10	97.0	97.7
4	TM24 (2)	–	*Methanobrevibacter gottschalkii* strain PG	96.9	–
4	TM42 (1)	–	*Methanobrevibacter millerae* strain ZA-10	96.7	–
4	TM49 (1)	–	*Methanobrevibacter millerae* strain ZA-10	97.2	–
5	TM6 (4)	GM11 (4)	*Methanobacterium aarhusense*	94.5	95.2
5	TM32 (1)	–	*Methanobacterium aarhusense*	95.1	–
6	TM40 (1)	–	*Methanobrevibacter gottschalkii* strain PG	96.5	–
6	TM23 (2)	GM29 (1)	*Methanobrevibacter millerae* strain ZA-10	96.1	96.9
7	TM35 (1)	GM13 (3)	Methanogenic archaeon mixed culture ISO4-G1	94.6	93.9
7	TM47 (1)	GM61 (1)	Methanogenic archaeon mixed culture ISO4-G1	93.8	93.9
7	TM53 (1)	–	Methanogenic archaeon mixed culture ISO4-G1	93.9	–
8	TM61 (1)	GM34 (1)	*Methanobrevibacter millerae* strain ZA-10	96.0	94.8
8	–	GM43 (1)	*Methanobrevibacter millerae* strain ZA-10	–	95.7
9	TM39 (1)	–	*Methanobrevibacter olleyae* strain KM1H5-1P	98.3	–
9	–	GM21 (2)	*Methanobrevibacter ruminantium* M1	–	98.8
9	–	GM39 (1)	*Methanosphaera stadtmanae*	–	98.7
10	TM56 (1)	GM15 (3)	*Methanobrevibacter millerae* strain ZA-10	97.7	98.0
10	–	GM19 (2)	*Methanobrevibacter smithii* strain PS	–	97.6
10	–	GM59 (1)	*Methanobrevibacter millerae* strain ZA-10	–	96.9
11	TM3 (7)	–	*Candidatus* Methanoplasma termitum strain MpT1	94.0	–
11	TM21 (2)	–	*Candidatus* Methanoplasma termitum strain MpT1	94.4	–
12	TM4 (6)	–	*Methanobrevibacter acididurans*	95.7	–
12	TM55 (1)	–	*Methanobrevibacter acididurans*	95.7	–
13	TM5 (5)	–	Methanogenic archaeon mixed culture ISO4-G1	94.4	–
13	TM33 (1)	–	Methanogenic archaeon mixed culture ISO4-G1	95.4	–
14	TM10 (4)	–	*Methanosphaera stadtmanae*	96.1	–
15	TM25 (2)	–	*Methanobrevibacter millerae* strain ZA-10	97.8	–
15	TM31 (1)	–	*Methanobrevibacter millerae* strain ZA-10	98.4	–
15	TM57 (1)	–	*Methanobrevibacter thaueri* strain CW	97.9	–
16	TM12 (3)	–	*Methanosphaera stadtmanae*	93.5	–
17	TM15 (3)	–	*Methanobrevibacter ruminantium* M1	96.6	–
18	TM18 (3)	–	*Methanobrevibacter ruminantium* M1	95.6	–
19	TM20 (2)	–	Methanogenic archaeon mixed culture ISO4-G1	96.6	–
20	TM41 (1)	–	Methanogenic archaeon mixed culture ISO4-G1	96.4	–
20	TM62 (1)	–	Methanogenic archaeon mixed culture ISO4-G1	95.7	–
21	TM54 (1)	–	*Methanobrevibacter smithii* strain PS	95.2	–
22	TM58 (1)	–	*Methanobrevibacter ruminantium* M1	97.1	–
23	TM60 (1)	–	*Candidatus* Methanoplasma termitum strain MpT1	94.1	–
24	TM30 (1)	–	*Methanobrevibacter smithii* strain PS	95.6	–

25	TM36 (1)	–	*Candidatus* Methanoplasma termitum strain MpT1	93.9	–
26	TM38 (1)	–	Methanogenic archaeon mixed culture ISO4-G1	96.1	–
27	TM43 (1)	–	Methanogenic archaeon mixed culture ISO4-G1	94.5	–
28	TM51 (1)	–	*Methanosphaera stadtmanae*	94.0	–
29	–	GM8 (4)	*Methanosphaera stadtmanae*	–	94.3
30	–	GM32 (1)	*Methanobrevibacter thaueri* strain CW	–	97.3
30	–	GM63 (1)	*Methanobrevibacter millerae* strain ZA-10	–	97.2
31	–	GM38 (1)	*Methanobrevibacter millerae* strain ZA-10	–	96.6
31	–	GM45 (1)	*Methanobrevibacter millerae* strain ZA-10	–	97.0
32	–	GM30 (1)	*Candidatus* Methanoplasma termitum strain MpT1	–	95.2
33	–	GM48 (1)	Methanogenic archaeon mixed culture ISO4-G1	–	89.3
34	–	GM49 (1)	*Methanobrevibacter millerae* strain ZA-10	–	96.6
35	–	GM54 (1)	*Methanobrevibacter millerae* strain ZA-10	–	97.6
36	–	GM56 (1)	*Methanobrevibacter olleyae* strain KM1H5-1P	–	96.5

[a]Phylotype sequences were obtained from MOTHUR program as unique sequences, while OTUs were generated by the MOTHUR program at 98% species level identity.
[b]Number of clones
[c]Percentage sequence identity to valid taxon
[d]The prefix TM and GM to represent rumen methanogen 16S rRNA gene sequences from Tibetan sheep and Gansu Alpine Finewool sheep clone libraries, respectively
