# Supplementary figures and images for: Rumen methanogen and protozoal communities of Tibetan sheep and Gansu Alpine Finewool sheep grazing on the Qinghai–Tibetan Plateau, China
Source: BMC Microbiol. 2018 Dec 13;18:212. doi: 10.1186/s12866-018-1351-0 (PMC6293568; doi:10.1186/s12866-018-1351-0)

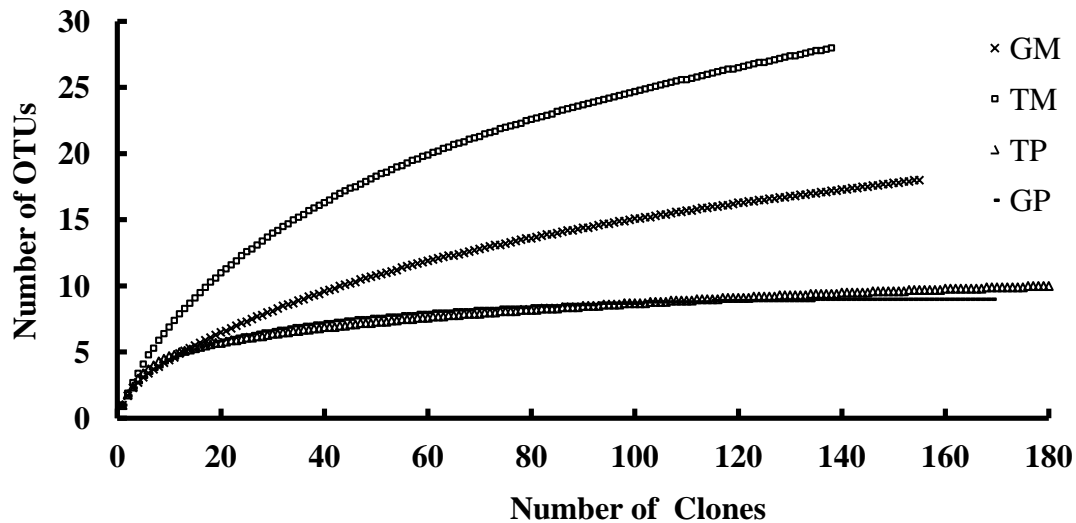

Supplement: Supplementary file 2 — Figure S1. Rarefaction curves generated for OTUs of 16S rRNA and 18S rRNA gene clones. The TM and GM stand for Tibetan sheep and Gansu Alpine Finewool sheep rumen methanogen 16S rRNA gene libraries, respectively. The TP and GP stand for Tibetan sheep and Gansu Alpine Finewool sheep rumen protozoal 18S rRNA gene libraries, respectively. (PDF 21 kb) [file 12866_2018_1351_MOESM2_ESM.pdf]
