# Supplementary material for: Rumen methanogen and protozoal communities of Tibetan sheep and Gansu Alpine Finewool sheep grazing on the Qinghai–Tibetan Plateau, China
Source: BMC Microbiol. 2018 Dec 13;18:212. doi: 10.1186/s12866-018-1351-0 (PMC6293568; doi:10.1186/s12866-018-1351-0)
Supplement: Supplementary file 3 — Table S2. Similarity values of rumen protozoa from Tibetan sheep and Gansu Alpine Finewool sheep from Qinghai-Tibetan Plateau, China. (PDF 52 kb) [file 12866_2018_1351_MOESM3_ESM.pdf]

Table S2 Similarity values of rumen protozoa from Tibetan sheep and Gansu Alpine Finewool sheep from Qinghai-Tibetan Plateau, China

OTU	Phylotype[a] (Clones[b])		Nearest valid taxon	Seq%[c]	
	TP[d]	GP[d]		TP	GP
1	TP3 (31)	–	*Entodinium furca monolobum* (AM158471)	98.3	–
1	TP15 (1)	GP1 (40)	*Enoploplastron triloricatum* (AM158462)	98.2	98.2
1	TP16 (1)	GP3 (22)	*Enoploplastron triloricatum* (AM158462)	98.2	98.2
1	TP17 (1)	GP5 (12)	*Enoploplastron triloricatum* (AM158462)	98.2	98.2
1	TP19 (1)	GP10 (2)	*Enoploplastron triloricatum* (AM158462)	98.5	98.3
1	TP24 (1)	–	*Entodinium furca dilobum* (AM158442)	98.7	–
1	–	GP15 (1)	*Enoploplastron triloricatum* (AM158462)	–	98.1
1	–	GP24 (1)	*Enoploplastron triloricatum* (AM158462)	–	98.2
1	–	GP25 (1)	*Enoploplastron triloricatum* (AM158462)	–	98.0
1	–	GP28 (1)	*Enoploplastron triloricatum* (AM158462)	–	98.2
1	–	GP33 (1)	*Enoploplastron triloricatum* (AM158462)	–	98.3
1	–	GP34 (1)	*Enoploplastron triloricatum* (AM158462)	–	98.2
2	TP4 (24)	GP8 (4)	*Epidinium caudatum* (U57763)	99.0	99.0
2	TP5 (11)	–	*Eremoplastron dilobum* (AM158472)	99.3	–
2	TP10 (1)	–	*Anoplodinium denticulatum* (AM158470)	99.4	–
3	TP7 (2)	GP4 (12)	*Diplodinium dentatum* (U57764)	99.6	99.3
3	TP9 (1)	GP18 (1)	*Diplodinium dentatum* (U57764)	98.6	98.5
3	TP12 (1)	GP12 (2)	*Anoplodinium denticulatum* (AM158470)	98.6	99.2
3	TP14 (1)	GP18 (1)	*Diplodinium dentatum* (U57764)	99.7	98.5
3	TP18 (1)	GP19 (1)	*Diplodinium dentatum* (U57764)	98.5	98.6
3	TP22 (1)	GP21 (1)	*Diplodinium dentatum* (U57764)	98.7	99.8
3	TP26 (1)	GP26 (1)	*Diplodinium dentatum* (U57764)	98.5	98.6
3	TP31 (1)	GP14 (1)	*Anoplodinium denticulatum* (AM158470)	99.2	99.3
3	–	GP29 (1)	*Diplodinium dentatum* (U57764)	–	99.3
3	–	GP36 (1)	*Anoplodinium denticulatum* (AM158470)	–	99.2
3	–	GP37 (1)	*Diplodinium dentatum* (U57764)	–	99.6
4	TP6 (2)	GP7 (9)	*Entodinium nanellum* (AM158449)	98.2	98.1
4	TP8 (2)	GP16 (1)	*Entodinium nanellum* (AM158449)	98.4	98.0
4	TP27 (1)	GP27 (1)	*Entodinium nanellum* (AM158449)	99.5	98.3
5	TP11 (1)	GP6 (10)	*Entodinium furca monolobum* (AM158471)	99.6	98.8
5	TP28 (1)	GP17 (1)	*Entodinium furca monolobum* (AM158471)	99.6	99.8
6	TP30 (1)	GP2 (27)	*Epidinium caudatum* (U57763)	99.2	99.8
6	–	GP20 (1)	*Epidinium caudatum* (U57763)	–	99.0
6	–	GP32 (1)	*Epidinium ecaudatum caudatum* (AM158474)	–	99.8
7	TP20 (1)	GP11 (2)	*Eudiplodinium maggii* (U57766)	99.8	99.7
7	–	GP22 (1)	*Eudiplodinium maggii* (AM158451)	–	99.9
7	–	GP23 (1)	*Eudiplodinium maggii* (U57766)	–	99.6
7	–	GP31 (1)	*Polyplastron multivesiculatum* (AM158458)	–	99.9

8	TP1 (44)	–	*Entodinium furca monolobum* (AM158471)	97.5	–
8	TP21 (1)	–	*Entodinium furca monolobum* (AM158471)	98.5	–
8	TP25 (1)	–	*Entodinium furca monolobum* (AM158471)	97.7	–
9	TP2 (41)	–	*Entodinium longinucleatum* (AB481099)	98.0	–
9	TP29 (1)	–	*Eudiplodinium maggii* (U57766)	97.8	–
10	TP13 (1)	–	*Dasytricha ruminantium* (U57769)	97.1	–
10	TP23 (1)	–	*Dasytricha ruminantium* (U57769)	98.3	–
11	–	GP13 (1)	*Dasytricha ruminantium* (U57769)	–	99.3
11	–	GP30 (1)	*Dasytricha ruminantium* (U57769)	–	99.0
11	–	GP35 (1)	*Dasytricha ruminantium* (U57769)	–	99.2
12	–	GP9 (2)	*Dasytricha ruminantium* (AM158463)	–	98.0

[a]Phylotype sequences were obtained from MOTHUR program as unique sequences, while OTUs were generated by the MOTHUR program at 98% species level identity.
[b]Number of clones
[c]Percentage sequence identity to valid taxon
[d]The prefix TP and GP represent rumen protozoal 18S rRNA gene sequences from Tibetan sheep and Gansu Alpine Finewool sheep clone libraries, respectively
