# Supplementary material for: Rumen methanogen and protozoal communities of Tibetan sheep and Gansu Alpine Finewool sheep grazing on the Qinghai–Tibetan Plateau, China
Source: BMC Microbiol. 2018 Dec 13;18:212. doi: 10.1186/s12866-018-1351-0 (PMC6293568; doi:10.1186/s12866-018-1351-0)
Supplement: Supplementary file 4 — Table S3. Genera of protozoa in the ciliate protozoan population of the Tibetan sheep and Gansu Alpine Finewool sheep rumen. (PDF 16 kb) [file 12866_2018_1351_MOESM4_ESM.pdf]

Table S3 Genera of protozoa in the ciliate protozoan population of the Tibetan sheep and Gansu Alpine Finewool sheep rumen

Genus	TP[a]		GP[a]	
	No. of clones	(%)Total clones	No. of clones	(%)Total clones
Entodiniomorphida	178	98.9	164	97.0
Entodinium	126	70.0	22	13.1
Enoploplastron	4	2.2	82	48.8
Epidinium	25	13.9	33	19.6
Eremoplastron	11	6.1	–	–
Anoplodinium	3	1.7	3	1.8
Diplodinium	8	4.4	19	11.3
Polyplastron	–	–	1	0.6
Eudiplodinium	1	0.6	3	1.8
Vestibuliferida	2	1.1	5	3.0
Dasytricha	2	1.1	5	3.0
Total	180	100	169	100

[a]The TP and GP stand for Tibetan sheep and Gansu Alpine Finewool sheep protozoal 18S rRNA gene libraries, respectively
